# Cholangiocyte Organoids: The New Frontier in Regenerative Medicine for the Study and Treatment of Cholangiopathies

**DOI:** 10.3390/jcm13061804

**Published:** 2024-03-21

**Authors:** Serena Babboni, Pier Giuseppe Vacca, Ludovica Simonini, Daniele Pezzati, Caterina Martinelli, Francesco Frongillo, Giuseppe Bianco, Emanuele Marciano, Giuseppina Basta, Davide Ghinolfi, Serena Del Turco

**Affiliations:** 1 Institute of Clinical Physiology, National Research Council, Via Moruzzi 1, 56124 Pisa, Italy; serenababboni@cnr.it (S.B.); ludovicasimonini@cnr.it (L.S.); lapina@ifc.cnr.it (G.B.); 2 Division of Hepatic Surgery and Liver Transplantation, University of Pisa Medical School Hospital, Via Paradisa 2, 56124 Pisa, Italy; piero93.vacca@gmail.com (P.G.V.); daniele.pezzati@ao-pisa.toscana.it (D.P.); caterina.martinelli@ao-pisa.toscana.it (C.M.); 3 Department of Surgery and Transplantation, Gemelli Hospital Foundation, Catholic University of the Sacred Heart, 20123 Rome, Italy; francesco.frongillo@policlinicogemelli.it (F.F.); giuseppe.bianco@policlinicogemelli.it (G.B.); 4 Division of Digestive Endoscopy, University of Pisa Medical School Hospital, Via Paradisa 2, 56124 Pisa, Italy; e.marciano@ao-pisa.toscana.it; 5 Istituto Italiano di Tecnologia, Smart Bio-Interfaces, Viale Rinaldo Piaggio 34, 56025 Pontedera, Italy

**Keywords:** organoids, cholangiocytes, biliary tree, cholangiopathies, regenerative medicine

## Abstract

Cholangiopathies include a group of chronic progressive disorders, affecting the cholangiocytes, the epithelial cells that line the biliary tree, leading to liver parenchymal fibrosis and eventually end-stage liver disease necessitating transplantation. Experimental modeling of these multifactorial cholestatic diseases faces challenges due to the lack of adequate experimental in vitro and in vivo models. A novel approach employs three-dimensional organoid systems that offer several advantages for modeling disease and testing drug response in vitro. Organoids mimic intercellular communication, replicate the architecture of organs, and maintain the cell’s original phenotype. Cholangiocyte organoids provide an in vitro model to study the pathogenesis and pharmacotherapeutic treatment of cholangiopathies and show great promise for regenerative therapies. In particular, patient-derived organoids allow personalized medicine approaches and the study of individual disease characteristics. This review highlights the significance of cholangiocyte organoid models in advancing our understanding of cholangiopathies and driving advancements in regenerative medicine strategies.

## 1. Introduction

Cholangiopathies are chronic progressive diseases that affect the biliary epithelium and can cause fibrosis and damage to the liver parenchyma, culminating in end-stage liver disease, which requires liver transplantation (LT) [1]. Cholangiopathies, such as primary sclerosing cholangitis (PSC), biliary atresia, and cholangiocarcinoma, are highly heterogeneous, characterized by unclear pathogenesis, and lacking well-defined therapeutic approaches to date [2]. In particular, PSC is characterized by biliary obstruction and damage to the liver itself, as well as by progressive biliary inflammation and fibrosis. The pathogenesis of PSC is not yet fully understood and appears to be caused by genetic, viral, and environmental insults, as well as unknown stimuli that contribute to the damage of cholangiocytes [3]. Evidence suggests the cholangiocyte is not a target of exogenous or endogenous stimuli but also likely a dynamic actor that, by the interaction with immune cells, endothelial cells, and mesenchymal cells, plays a key role in the progression of the disease [4].

Because of a multifactorial etiology and multiple different features, cholestatic diseases have limited experimental models. In vitro cell culture models are key in liver research to supplement the lack of human samples or in vivo animal models [5,6]. However, conventional two-dimensional monolayer cell cultures lack the representation of intercellular cell-to-cell interaction, and primary human cholangiocytes are difficult to isolate and de-differentiate after a few passages [7]. On the other hand, three-dimensional cell culture systems, such as organoids, can effectively mimic cell-to-cell interaction, architecture, communication, and microenvironment among liver different cells. Organoids derived from primary human cholangiocytes have the advantage of preserving the original phenotypes of the cells; patient-derived cholangiocytes and cholangiocarcinoma organoids provide a tool for disease modeling purposes and offer an interesting platform for drug screening applications [8]. The utilization of healthy donor-derived human cholangiocyte organoids highlights their potential applications in tissue engineering and regenerative medicine and have shown the potential to restore damaged biliary epithelia in preclinical models [9].

This review synthesizes the insights into cholangiopathies and investigates cholangiocyte organoid models both as advanced tools for understanding cholangiopathies and as a cornerstone strategy in the field of regenerative medicine. A comprehensive analysis of the challenges that have to be overcome for organoids’ clinical application is also conducted.

## 2. Cholangiocytes and the Biliary Tree

**Figure 1:**
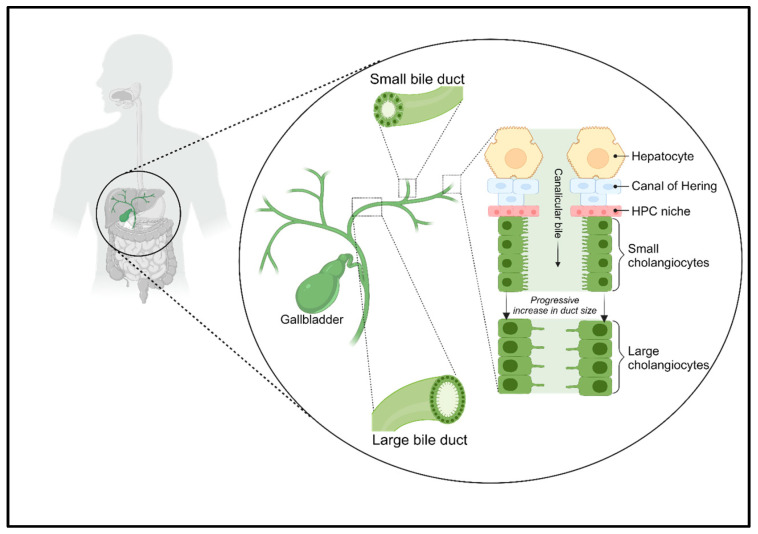
The biliary tree complex. The biliary tree consists of small and large bile ducts, which are responsible for the active transport of electrolytes and solutes through cholangiocytes, which alter the flow of canalicular bile. The Hering canals connect hepatocytes to cholangiocytes and are lined by a niche of HPCs.

The biliary tree is a complex network of tubular structures, or bile ducts, which begins with the Hering canals in the hepatic lobules and progressively merges into a system of interlobular, septal, and major ducts that all together form the extrahepatic bile ducts. The complex is divided into two compartments, intrahepatic and extrahepatic, which differ both anatomically and functionally [10] (Figure 1).

Hering canals connect the hepatocellular canalicular network that carries primary bile from the liver to the gallbladder, where it is stored, and which ends in the Vater Ampulla, from which the bile is poured into the small intestine where it contributes to the digestion of lipids [11,12]. Hering canals are the site of the hepatic stem cell niche [13]. Instead, the extrahepatic biliary network presents a niche of hepatic progenitor cells (HPCs), which differs from that present in the Hering canals, because the intra- and extrahepatic biliary trees have different embryonic origins [14].

Cholangiocytes are ciliated and highly specialized epithelial cells that line the intra- and extrahepatic bile ducts. They play a key role in liver repair, innate immunity, and progression of cholangiopathies [10]. They represent the first line of defense in the innate immunity of the liver; moreover, they can be both the target of immune-mediated aggression or the initiators of an inflammatory reaction that progresses to adaptive immune activation [4]. The contribution of biliary epithelial cells to liver immune responses was thought to be limited to the secretion of immunoglobulin (Ig) A in bile, but it is clear at present that the role of cholangiocytes in immune response is much more complex [15].

Cholangiocytes within various compartments of the biliary tree exhibit a distinct morphology. The small cholangiocytes line the Hering canals and distal branches of the biliary tree, featuring a cuboidal shape with a round basal nucleus. They exhibit rapid reactivity to liver and/or biliary damage [16]. On the other hand, large cholangiocytes line the larger-diameter ducts, displaying a cylindrical shape. These cells are involved in secretory functions and possess transport capacity, mediating processes such as alkalization, hydration, and the modification of bile [17].

The apical surface of cholangiocytes has a non-mobile primary cilium, which functions as a mechanosensor, chemosensor, and osmosensor. The cilia direct the bile flow and, by bending, activate the calcium ions (Ca^2+^) channel, allowing the influx of Ca^2+^ into the cell [11]. These structures can be involved in cell proliferation and senescence, in the activation of progenitor cell compartments, and in regeneration and development.

The extracellular vesicles present in the bile can bind to cilia and have been shown to inhibit the proliferation of bile duct cells, promoting a quiescent state of the biliary system under normal conditions [15]. Moreover, recent research has shown that exosomes (small extracellular vesicles ranging in size from 50 to 200 nm, containing genetic material, such as DNA, mRNA, and various types of non-coding RNAs) are promising diagnostic tools for cholangiocarcinoma and gallbladder carcinoma, as they are easily and rapidly accessible [18]. Circulating non-coding RNAs, found within exosomes, can play a significant role as effective biomarkers for the diagnosis of various diseases. Therefore, the analysis of exosomes and their genetic contents could represent an innovative approach to the early and precise diagnosis of various pathological conditions.

Biliary epithelial cells, thanks to their ability to secrete ions in a polarized way and their selective permeability to solutes and water, actively maintain hepatic homeostasis. In addition, the biliary epithelium acts as a barrier against the back diffusion of xenobiotics, toxic metabolites, and bile salts from the bile to the interstitial tissue [19].

The cells of the smaller branches of the bile ducts have different and specific biological properties, such as phenotypic plasticity, the ability to react to liver damage and behavior as the progenitors of hepatocytes, and they are awakened to varying degrees only after liver damage [10].

## 3. Biliary Injury

**Figure 2:**
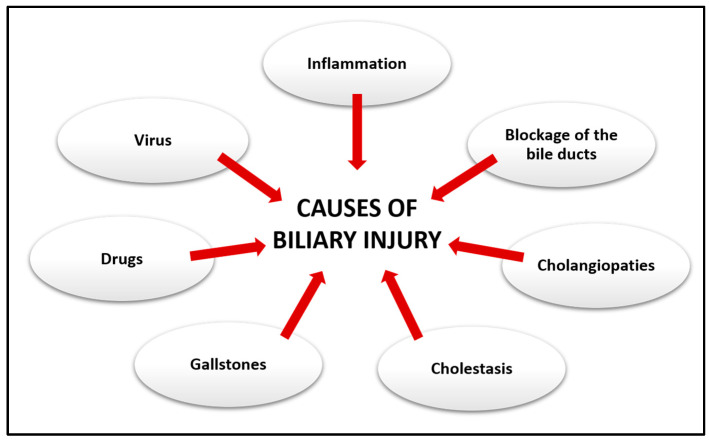
Causes of biliary injury. Damage to the biliary system can result from a variety of different conditions.

The damage to the biliary tree can be various in nature (Figure 2). Most of the disorders that cause biliary pain are due to calculi, that is, the formation of stones within the bile ducts and in the gallbladder causing choledocholithiasis and cholelithiasis [20]. These, in turn, can cause biliary colic and cholecystitis, scilicet inflammation of the gallbladder, which can be acute if it progresses in a few hours or chronic if it evolves for a longer time [21]. Furthermore, blockage of the bile ducts can also lead to inflammation of the bile ducts thus causing acute cholangitis. However, the blockage or slowing of the flow of the bile ducts, known as cholestasis, can also be caused by tumors or strictures following viral infections [20,22].

Cholestasis in turn can progress into chronic liver disorders affecting cholangiocytes, known as cholangiopathies, which can result from proliferative, fibrotic, genetic, immune-mediated phenomena that can cause portal hypertension and progressive periportal fibrosis [23]. The different distributions of cholangiopathies along the biliary tree can be explained by the different types of damage that can affect cholangiocytes [24]. Cholangiopathies can generally be classified into immune-mediated diseases, infectious, genetic, inflammatory, and fibrosis, which lead to the development of primary biliary cholangitis (PBC), PSC, IgG4-related sclerosing cholangitis (ISC), and biliary atresia (BA) [19,25,26].

Cholangiocytes are the target of various stimuli of innate and adaptive immune responses, ischemia, cholestasis, and xenobiotics [27,28]. Their activation causes an increase in pro-inflammatory and pro-fibrotic mediators, and the recruitment of immune, vascular, and mesenchymal cells, which all together contribute to the development of biliary fibrosis, which can ultimately evolve into cholangiocarcinoma [4,29].

### 3.1. Cholangiopathies

PBC is a chronic and progressive disease mainly observed in females. Its incidence is 1–2 per 100,000 population per year, prevalence is 1 over 1000 in women older than 40 years, and is strongly associated with autoimmune syndromes, such as Hashimoto’s thyroiditis, Sjögren’s disease, celiac disease, or systemic sclerosis. PBC is characterized by anti-mitochondrial (AMA) or specific anti-nuclear antibody (ANA) positivity. Ninety per cent of PBC patients show AMA positivity. Histology of PBC shows the typical florid duct lesions and destruction of intralobular bile ducts. The pathogenesis is poorly understood; however, autoimmunity is likely involved [30].

Instead, PSC is mainly observed in men with a median age at diagnosis of 41 years; incidence is 0–1.3 cases for 100,000 persons per year. It is strongly associated with inflammatory bowel disease (IBD) and gallbladder and colorectal cancers. It represents the major risk factor for cholangiocarcinoma [31]. Magnetic Resonance Imaging and Endoscopic Retrograde Cholangiopancreatography (ERCP) show the characteristic strictures that confirm the diagnosis. The structures involve either the entire biliary tract (95%) or only the small ducts [32]. Pathogenesis is still unknown. The role of activated T cells has been proposed as a potential cause. Nevertheless, the presence in the liver parenchyma of microbial antigens could be involved in the early senescence of hepatocytes.

ISC is an uncommon variant of PSC. It has been associated with a worse prognosis, without IBD, even though the etiology is still not clear. Serum IgG4 is elevated both in ISC and in PSC, but ISC is associated with tissue IgG4 deposits and inflammatory disease of other glands, such as pancreatitis or sialadenitis. Lastly, ISC shows a good clinical response to glucocorticoid treatment, but the relapse percentage is frequent [33]. Table 1 shows the epidemiological data of the most widespread cholangiopathies.

### 3.2. Biliary Complications Post-LT

Cholangiopathies are a frequent indication for LT. The European Liver Transplant Registry (ELTR) reported that 13,241 LTs were performed for cholestatic disease in the last 50 years (10% of the total): 44% due to PBC, 44% due to PSC, and the remaining due to secondary biliary cirrhosis. These percentages remained stable over the past 15 years, except for a slight increase in PSC [34]. About 40% of patients with PSC undergo an LT. However, a recurrence is observed in 10 to 40% of cases, leading to re-transplantations in up to 50% of cases. Acute or chronic rejection is frequent (39–71%) requiring a high immunosuppressive regimen. PSC remains a clinical and surgical challenge, with a 1-year survival rate of 85% and a 5-year survival rate of 72% [2,3,23,35].

Furthermore, the extension of the criteria for liver donation, with the inclusion of elderly donors and donors after cardiac death (DCD), caused an increased risk of developing complications that could lead to graft failure [36]. Among the most common complications are biliary complications, which are increasingly the cause of morbidity and mortality after transplantation, and the most relevant ones are: anastomotic (AS) and non-anastomotic stenosis (NAS).

NAS occurs due to irregularities of the biliary tree and represents the most common complication [37]. The origin is often multifactorial, and multiple causes may overlap with damage to the biliary system, such as bile duct injury and subsequent fibrosis and the gross narrowing of donor bile ducts. NAS can be further classified as an ischemic-type biliary lesion (ITBL), which is associated with arterial stenoses or thrombosis and ischemic cholangiopathy (IC), where normal vascular flows are present [38]. IC is characterized by an increase in cholestasis indices and bilirubin, often associated with fever and abdominal pain [39]. Strategies aimed at preventing IC include the implementation of dual (portal and arterial) perfusion at perfusion at procurement and ex-situ machine perfusion preservation, which are extremely relevant [36,40,41]. The current main explanations for a higher rate of NAS in DCD transplants are ischemia-reperfusion injury (IRI), immune processes, and bile salt toxicity that damage cholangiocytes [42]. DCD livers undergo a period of warm ischemia in the donor, which, combined with cold storage and other risk factors, makes the liver more susceptible to the development of ITBL [36,43]. IRI can be classified as a primary ischemia injury that affects the bile ducts during the transplantation stages, secondary ischemia that can occur after transplantation due to damage to the peribiliary vascular plexus, and insufficient regeneration of the biliary epithelium [36,44].

Primary injury can occur at various stages during the transplantation procedure. An extended warm ischemia time in the donor, particularly when combined with additional cold storage, represents a key risk factor for the development of biliary strictures [44,45,46]. Following reperfusion, the damage is aggravated as accumulated oxygen-containing reactive species (ROS) and damage-associated molecular patterns (DAMPs) proceed to activate the immune system causing necrosis and apoptosis. It has been shown that cholangiocytes are more susceptible to IRI than hepatocytes because of slower ATP regeneration, higher ROS production, and lower concentration of glutathione, which has an antioxidant action [47,48].

After that, DCDs undergo a second warm ischemia when the organ is harvested from ice and placed in the recipient’s abdomen, due to portal reperfusion, which is low in oxygen saturation, does not contribute sufficiently to biliary perfusion, and each additional minute of warm ischemia increases the risk of ITBL [49,50]. In addition, hepatic steatosis of the transplanted liver also contributes to secondary ischemia damage, as it causes the swelling of lipid-laden hepatocytes that causes the impairment of micro-circulation and increases the risk of developing biliary complications [44,51].

The IRI of the bile ducts has long been considered the main determinant of the development of ITBL. However, several clinical studies have shown that extensive injury and loss of the biliary epithelium can be found in more than 90% of transplanted livers, and only a minority of these develop post-transplant cholangiopathy [44,52]. This brings us back to the hypothesis that the insufficient regeneration of the biliary epithelium, rather than the initial amount of injury, determines whether a liver donor develops post-transplant cholangiopathy [52]. Currently, the treatment of post-transplant cholangiopathy consists of the use of antibiotics, endoscopies, resection of extrahepatic bile ducts, and liver re-transplantation, but these are often challenging and unsuccessful.

In recent years, attention has shifted to the use of mechanical perfusion as an emerging strategy to counteract IRIs [8,52]. Studies have indicated that the use of the normothermic machine perfusion (NMP) in cases of DCDs is associated with a reduced incidence of post-transplant biliary damage, due to the supply of oxygen and nutrients and consequent reduction in ischemic damage in the bile duct [36]. Using DCD livers from pigs, the positive effect of NMP on biliary damage and regeneration has been demonstrated [53,54]. But, it was also confirmed by the de Jong study [55], which found an increase in the proliferation of cholangiocytes during NMP and better preservation of the peribiliary glands (PBGs) containing progenitor cells that differentiate into mature cholangiocytes for biliary regeneration. Compared to NMP, hypothermic perfusion (HMP) also presents advantages. Multiple studies have demonstrated that HMP significantly reduces IRI. Furthermore, it appears to “resuscitate” mitochondrial function, thereby decreasing the formation of ROS and the activation of the immune system [42,44]. Additionally, studies on pigs regarding HMP showed the enhanced preservation and protection of the bile ducts, ultimately leading to an improved hepatobiliary function [56]. Moreover, MP can act as a platform for the direct release of therapeutic agents to organs before transplantation and for testing new therapeutic approaches and their effectiveness in repairing liver damage [36,56,57,58,59].

A new strategy would involve transplanting cholangiocyte organoids directly into intrahepatic ducts before organ transplantation, during machine perfusion (MP) [60]. Cholangiocytes play an important role in the etiopathogenesis of post-transplant cholangiopathies [42,49,61], and given the high incidence of biliary system disorders following transplantation, the use of cholangiocyte organoids has been proposed. The study by Sampaziotis et al. [8] highlights the high plasticity of cholangiocytes: cells taken from different regions of the biliary tree contain different transcriptional profiles, but cholangiocytes lose these differences by allowing cells from one region to repair a different region of the biliary tree.

## 4. Regeneration in Response to Biliary Damage

**Figure 3:**
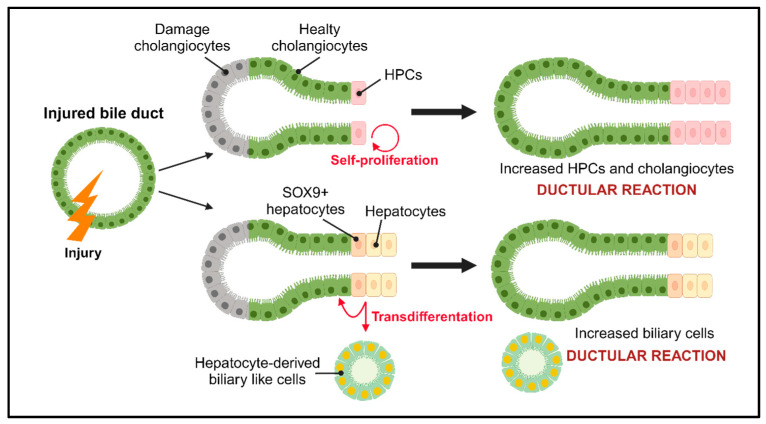
Regeneration after biliary injury. After damage, cholangiocytes and HPCs are activated to compensate for the damaged cells through the ductal reaction. Depending on the type of damage, two mechanisms are implemented: self-proliferation in which the populations of HPCs and cholangiocytes increase, and the transdifferentiation of hepatocytes into biliary-like cells and/or cholangiocytes. HPCs: hepatic progenitor cells; SOX9: SRY-box transcription factor 9.

In response to biliary damage, cholangiocyte proliferation is activated to maintain normal homeostasis of the biliary tree. In physiological conditions, small cholangiocytes are quiescent, but in case of liver damage, they activate a marked proliferation as part of the hepatic reparative complex [62] (Figure 3). Moreover, cholangiocytes are involved in cell cycle phenomena that maintain tissue homeostasis in the biliary system through modulators of apoptosis and senescence, and damage to cholangiocytes can lead to cholangiopathies [19].

Based on the type of damage, a specific population of cholangiocytes is activated to start proliferating. The proliferation of cholangiocytes can be divided into three types: the “typical” type results in an increase in the number of intrahepatic bile ducts deriving from older pre-existing ducts [63]; the “atypical” type occurs in chronic liver injury as well as alcoholic liver disease and chronic extrahepatic biliary obstruction, and results from the transdifferentiation of hepatocytes into cholangiocytes [64]; and the last type is type III and it is the first step towards carcinogenesis in the liver, which leads to disorganized proliferation and distorted liver architecture [65].

Inflammation, caused by the activation of cholangiocytes following damage, is at the basis of the biliary repair process and biliary fibrosis known as the ductal reaction (DR), which involves inflammatory cells, HPCs, and activated cholangiocytes [66]. The DR is defined as a bile duct hyperplasia commonly observed in liver diseases and the cellular phenotypic profile that characterizes it is influenced by the location of the liver lesion and the etiology of the disease that causes the lesion [66,67]. Therefore, in case of damage to the biliary tree, the reaction is characterized by the proliferation of cells with a biliary profile, while damage to hepatocytes causes a proliferation of cells with a hepatocytic profile [67,68].

HPCs are stem cells that begin to proliferate and expand rapidly following severe liver damage and have bidirectional differentiation potential as they can differentiate into hepatocytes and/or cholangiocytes [69]. There are two distinct populations of progenitor cells: hepatic progenitor cells (HPCs) and biliary tree progenitor cells (BTPCs).

HPCs are found in the smallest branches of the biliary tree, in the Hering canals and bile ducts. Their activation is associated with the appearance of the ductular reaction and in the context of cholangiopathies support the renewal of cholangiocytes that are compromised in their proliferative abilities [70].

BTPCs, which are found in the PBGs of the large intrahepatic and extrahepatic bile ducts [71], proliferate in response to biliary damage to give rise to the progeny of cholangiocytes [72]. Furthermore, damage to PBGs at the time of transplantation is a risk factor for the development of biliary complications. Therefore, it is plausible to think that post-transplant cholangiopathies are determined by the effective regenerative capacity of the bile ducts, rather than the amount of epithelial damage [73].

In case of cholestatic liver diseases, such as PSC or PBC, an “atypical” proliferation occurs where hepatocytes transdifferentiate into cholangiocytes and/or cholangiocyte-like cells, contributing to functional repair and regeneration in liver damage. This transdifferentiation is a result of cellular reprogramming observed through the expression of biliary transcription factors and other specific markers [64,74].

## 5. “Old” Therapies for the Regeneration of the Biliary Tree

Over the years, several treatments have been designed to prevent the progression of cholangiopathies, but unfortunately, they have failed and almost always the only solution is LT (Table 2).

Ursodeoxycholic acid (UDCA) has been, for a long time, and is still the gold standard for the treatment of cholangiopathies [25,26]. It is a hydrophilic bile acid naturally present in bile, which is effective in preventing the progression of inflammation and fibrosis when taken early in the disease [75]. However, this treatment appears to be ineffective in 40% of patients [76]. UDCA efficacy in PSC is still debated, as it determines an improvement in blood chemistry without increasing survival. According to other studies, UDCA may even determine a worsening of the prognosis, with a higher incidence of cirrhosis and cholangiocarcinoma and the need for LT.

In general, even if UDCA is the main treatment for these pathologies, their effectiveness is limited, and also in PBC—where the evidence is stronger—there are no differences in symptoms, liver-related mortality, or transplant-free survival [77].

Obeticholic acid (OCA), 24-Norursodeoxycholic acid (*nor*UDCA), and antibiotics are widely used, but they are still under study or have limitations/complications, like the risk that elevated serum fibroblast growth factor 19 (FGF19) levels lead to the development of hepatobiliary malignancy and the high dosage that is toxic [31,32,78,79].

It is not indicated in patients with decompensated cirrhosis or portal hypertension, but only in patients with Child–Pugh Class A. The dosage must be titrated progressively and is often not tolerated by patients due to the onset or worsening of itching, fatigue, nausea, and headache [77]. The data regarding the long-term impact on survival in patients treated with OCA are limited.

Other treatments are the application of immunosuppressants, glucocorticoids combined with UDCA, and B-cell depletion, but potential treatments, such as signal regulatory protein 1 and 4 (S1RP1, S1RP4) agonists and NADPH oxidase 1 and 4 (NOX1, NOX4) inhibitors, are under clinical evaluation [75]. Glucocorticoid and azathioprine represent the first-line therapy, however other antimetabolite drugs or calcineurin inhibitors can be administered. The response rate is high, but relapse is frequent. Transplantation may be necessary in cases of severe acute hepatitis [80].

**Table 2:** Mechanisms of action of some treatments for cholangiopathies.

Treatment	Mechanism	References
UDCA	Protection of biliary epithelial cells and mitochondrial integrity, reduction in pro-inflammatory cytokines.	[76,79]
OCA	FXR agonist that suppresses bile acid synthesis, inflammation, and hepatic fibrosis, and induces the endogenous synthesis of FGF19. It is a promising potential therapy for PBC patients.	[81,82]
*nor*UDCA	Increases resistance to biliary damage induced by bile acids and has a pleiotropic effect on inflammation, apoptosis, and fibrosis.	[83,84]
Antibiotics	Improvement in liver biochemistry observed with vancomycin, metronidazole, azithromycin, and minocycline.	[78]

The ineffectiveness of the treatments used at present leads to the only therapeutic option of a transplant [85]. So, the lack of definitive therapies and the high cholangiocytic disease incidence have prompted the exploration of new alternatives, such as the utilization of organoids to repair biliary damage.

## 6. Cholangiocyte Organoids as a New Strategy for the Regeneration of the Biliary Tree

The failure of regeneration and limited treatments for advanced liver disease highlight the urgent need for new strategies in regenerative approaches that activate the body’s natural repair mechanisms and explore options such as cell-based therapies or bioengineered tissue for liver replacement.

The strategies of regenerative medicine, for the liver as well as for other organs, can be captured through the R3 paradigm: replacement, regeneration, and rejuvenation [6] The replacement strategy involves LT, the only clinically available regenerative medicine therapy in end-stage liver disease. However, this paradigm helps guide the development and implementation of complementary strategies, such as cell-based therapies (e.g., liver organoids) and bioengineered tissues. In contrast, regeneration involves the delivery and engraftment of stem cells or progenitor cells that then undergo growth and differentiation in vivo (e.g., stem cell transplant or stem cell-coated stents) [9,10,11]. Lastly, rejuvenation involves inducing tissue self-renewal through the activation of endogenous stem cells (e.g., gene therapy or exosome delivery) [14].

The hepatic, pancreatic, and biliary Organoid Consortium recently published a consensus document that defines an organoid as a “three-dimensional structure derived from (pluripotent) stem cells, progenitor, and/or differentiated cells that self-organize through cell–cell and cell–matrix interactions to recapitulate aspects of the native tissue architecture and function in vitro” [86]. The organoids are then classified according to single or multiple germ lines in epithelial, multi-tissue, and multi-organ organoids, and also subclassified according to cell type of origin.

### 6.1. Generation of Cholangiocyte Organoids

**Figure 4:**
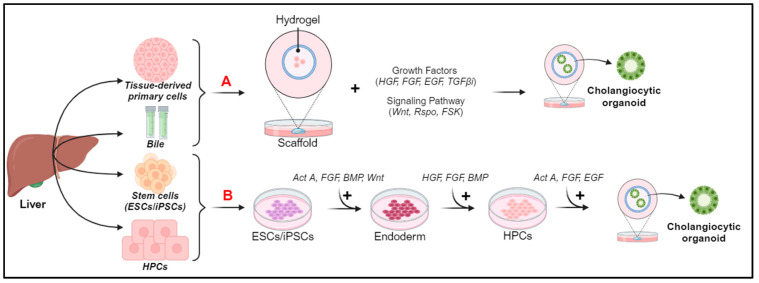
Sources and generation of cholangiocyte organoids. (**A**) Organoid generation from tissue-derived primary cells and bile. (**B**) Organoid generation from ESCs/iPSCs and HPCs. Act A, Activin A; BMP, bone morphogenic protein; EGF, epidermal growth factor; ESCs, embryonic stem cells; FGF, fibroblast growth factor; FSK, forskolin; HGF, hepatocyte growth factor; HPCs, progenitor cells; iPSCs, pluripotent stem cells; OSM, oncostatin M; TGFβ1, transforming growth factor beta inhibitor; TNFα, tumor necrosis factor alpha.

Cholangiocyte organoids can derive from the epithelial cells of different compartments of the biliary tree (intrahepatic, extrahepatic, and bile); therefore, a nomenclature has been proposed that allows us to identify the organoids based on their origin: intrahepatic cholangiocyte organoids (ICOs), gallbladder cholangiocyte organoids (GCOs), extrahepatic cholangiocyte organoids (ECOs), and cholangiocyte organoids derived from bile (BCOs) [60]. All of these structures share similar phenotypic characteristics when grown, but they have different identities based on the location and composition of the bile [87]. It has been observed that cholangiocytes have a plastic identity and lose some characteristics of the subpopulation of origin to assume a single common organoid identity, but if exposed to different bile concentrations and compositions, they can regain the identity of the original location [8,88]. Cholangiocyte organoids can be derived from both healthy and diseased individuals and can be generated from a variety of sources, including stem cells (iPSCs), organ-derived primary tissues, or body fluids, such as bile [86] (Figure 4).

Organoids derived from tissue-derived primary cells have greater stability and ease of propagation than organoids derived from iPSCs, but require access to the primary tissue, and this is not always possible [89]. For organoid generation from tissue-derived primary cells or bile (a schematic representation is shown in Figure 4A), the Scaffold technique that exploits the extracellular matrix (ECM)-based hydrogel or Matrigel is used [90,91]. It is commercially available, and, through different growth factors in the culture medium and signaling pathways, recreates a bioactive micro-environment in which the cells differentiate and generate spherical structures and finally mature into cholangiocytes or hepatocyte organoids [60,90].

Pluripotent stem cells (iPSCs), do not require access to primary tissue, as they allow us to easily obtain cholangiocytic organoids with minimally invasive procedures from different materials, such as the blood, urine, or skin [92]. The resulting cells differentiate into hepatoblasts, which can give rise to the monolayer of HPCs [93] (Figure 4B). Then, single-cell or multicellular approaches can be used to obtain cells that exhibit the key functions of mature cholangiocytes [92]. The multicellular approach involves the interaction of HPCs with OP9 cells, a stromal cell line that expresses the Notch ligand (of the bile duct regeneration pathway), which form cells that express primary biliary and cilia functions [70]. The single-cell approach is less variable, as it relies only on key factors of biliary regeneration pathways (such as Wnt, Notch, and TGFβ), which lead to the formation of cells expressing markers (cytokeratin19, SOX9, and CFTR), primary cilia, and stimuli secretors [94]. Despite the presence of many features of mature cholangiocytes, iPSC-derived cholangiocyte organoids are characterized by incomplete maturation, some fetal characteristics, and the genetic instability associated with iPSC [48]. To overcome this challenge, Ogawa et al. [70] devised a monolayer-based differentiation strategy, enabling the generation of a significant number of mature and ciliated cholangiocytes from various pluripotent stem cell lines, thus paving the way for exciting opportunities to develop targeted cellular therapies for regenerating compromised and/or diseased bile ducts in patients with cholangiopathies [95].

Cholangiocyte organoids that are derived from HPCs are an exciting area of research in the fields of regenerative medicine and modeling liver diseases and their use to produce cholangiocyte organoids has several advantages: these cells have significant proliferative potential, which means they can multiply quickly in vitro and provide a generous source of material for experiments; the genomic stability is crucial in ensuring the consistency of results in organoid cultures; and the intrinsic re-differentiation capacity of HPCs within organoids guarantees the flexibility to transform into various cell types, including cholangiocytes, and this feature enhances the organoids’ ability to reflect the complexity and heterogeneity of liver tissue [96,97].

Primary cholangiocytes can be grown using two main complementary platforms, based on canonical or noncanonical Wnt signaling, resulting in a different cell phenotype. Wnt seems to be a master regulator of a mature versus stem cell phenotype [98,99]. Cells grown in the canonical Wnt signaling condition have a stem cell-like phenotype and can differentiate toward both the hepatic and biliary lineage, but do not fully recapitulate the functions of mature cholangiocytes or hepatocytes in vitro. Primary cholangiocytes grown in conditions based on noncanonical Wnt signaling give rise to mature primary cholangiocyte organoids in the long term while maintaining genetic stability, expression of key mature biliary markers, and cholangiocyte functions in vitro, maintaining their plasticity.

### 6.2. Applications of Cholangiocyte Organoids

**Figure 5:**
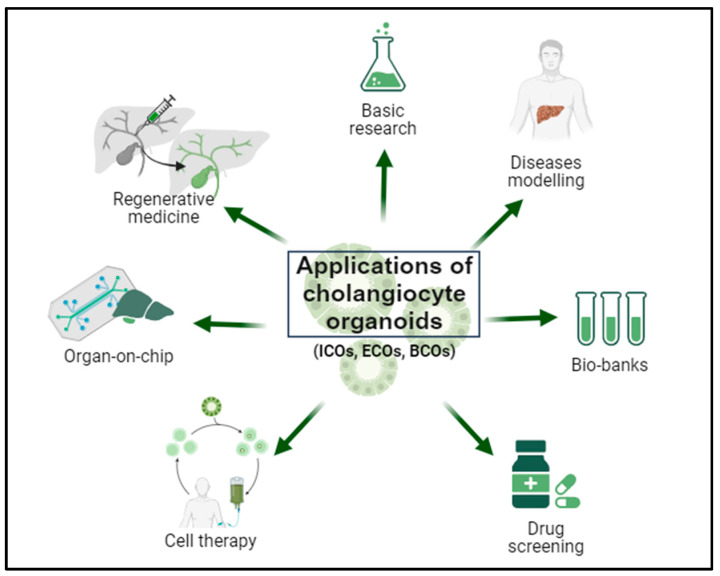
Biomedical applications of cholangiocyte organoids. Cholangiocyte organoids can be derived from intrahepatic bile duct biopsies (intrahepatic cholangiocyte organoids; ICOs), extrahepatic bile duct biopsies (extrahepatic cholangiocyte organoids; ECOs), and bile samples (bile-derived cholangiocyte organoids; BCOs). Organoids offer a broad spectrum of applications, including basic research on liver physiopathology, disease modeling, biobank establishment, pharmacological screening, implementation of personalized therapies, utilization in organ-on-chip systems, and application in regenerative medicine.

The use of cholangiocyte-derived organoids in the context of cholangiopathies and bile system disorders holds great promise. This is attributed to the regenerative potential of bile epithelia distributed throughout the liver and the unique plasticity of cholangiocytes, endowing these cells with a unique potential for tissue repair while retaining the functions and characteristics of the original tissue [8]. Fundamentally, organoids are well-suited for basic research on liver pathophysiology, disease modeling, pharmacological treatment assessment, and the development of personalized treatments, as well as applications in regenerative medicine for repairing deficits in the bile epithelium [100]. Additionally, the three-dimensional structure of organoids offers the opportunity to develop multi-organ systems and evaluate the contribution of various organs through platforms like the liver on chip (Figure 5).

#### 6.2.1. Basic Research and Disease Modeling

Cholangiocyte organoids can be employed in basic research, enabling the study of liver cell differentiation, including the development and maturation stages of cholangiocytes [60]. Furthermore, 3D cultures derived from healthy patient samples allow the modeling of liver diseases, including cholangiopathies, by recreating the physiological microenvironment (including cell–cell and cell–ECM interactions) [7].

For instance, bile-derived organoids (BCOs) have been identified as a novel method for studying the pathogenesis and therapy of cholangiopathies, such as PSC. While tissue-derived organoids are limited by sample availability (usually collected during transplants), BCOs are easier to obtain since bile can be regularly collected [88,101]. Additionally, they exhibit a biliary phenotype and altered expression of genes associated with immune regulation [102]. BCOs from PSC patients, when stimulated with the pro-inflammatory cytokine IL-17A, were observed to secrete high levels of chemokine CCL20, confirming its key role in biliary duct damage in patients with chronic liver inflammatory diseases [103]. This discovery is valuable as it opens avenues for studying pharmacological therapies for these organoids, making them more amenable to susceptibility testing. However, while BCO technology presents limitations due to the not fully matured origin epithelia and the ability to obtain them only from patients with biliary duct stenosis, these organoids preserve more properties of the biliary tree compared to iPSCs; hence, further comparative studies are warranted [104].

Moreover, Chen et al. [105] utilized primary cholangiocytes isolated from mouse bile ducts and decellularized liver scaffolds to develop functional ductal organoids (FDOs) and construct a network structure resembling a biliary tree. The study demonstrated that cholangiocytes in FDOs could have a future in clinical therapy, as they can be used for disease modeling and generating bioengineered livers [105].

The simulation of pathological conditions is a widely explored area for organoid use, even enabling the replication of congenital and hereditary diseases through genetic manipulation techniques [100]. Additionally, organoids derived from bile ducts open up the possibility of creating human organoid biobanks (healthy or diseased), thus enabling future studies on diseases, pharmacological screenings, and personalized medicine [9].

#### 6.2.2. Drug Screening and Organ-on-Chip System

Organoids derived from the cells of diseased individuals can be used to develop personalized gene therapies [90]. In fact, cells within the organoid can be employed to test the effects of drugs, thus aiding in identifying potential therapies and possible hepatotoxic effects they can cause, also thanks to the secretion of enzymes into the bile, which are useful for the hepatic metabolism of many hydrophobic drugs [7,106,107].

The lack of adequate in vitro models prompted the group of Shi et al. to use intrahepatic cholangiocyte organoids to recreate necroptosis, a common mode of programmed cell death in cholangiopathy [108]. They demonstrated that ICOs can serve as a useful platform for the in vitro study of biliary cytotoxicity and preclinical assessment of drugs, with significant implications for the development of therapies for cholangiopathies.

Recently, organ-on-chip (OOC) technology has been developed, representing an innovative preclinical system for the in vitro evaluation of human organ responses to anti-tumor therapies [109]. Combined with organoid cultures, it can enable in vivo drug screening [110]. The advantage of chips lies in their ability to recreate the multicellular structure, chemical gradients, vascular systems, and mechanical properties of human organs [111,112].

Organoids for tumor studies are limited as they lack components of the immune system, can be contaminated by normal organoids, and require components necessary for organoid development (growth factors, extracellular matrix, and serum). On the other hand, OOCs also presents various limitations due to their lower 3D complexity compared to organoids and longer processing times [113,114]. The combination of organoids and OOCs can allow the integration and combination of different tumor cell lines and immune cells, recreating and assessing immune microenvironment interactions in a more physiologically relevant and comprehensive manner. This has the potential to predict targeted therapy for the patient, revolutionizing preclinical tools in precision medicine [115,116].

#### 6.2.3. Regenerative Medicine

The incidence of liver diseases is on the rise globally, and currently, LT is the only therapeutic option for end-stage liver diseases. The development of regenerative medicine techniques holds great potential, with the biliary system being a particularly interesting target due to its significant regenerative potential throughout the liver and its minimally invasive accessibility through ERCP [117,118]. Organoid culture allows the propagation of highly functional cells that retain their original functions and demonstrate the ability to engraft and regenerate the liver or bile ducts [119]. Notably, organoids adapt well to large-scale expansion, enabling the generation of autonomous mini-liver structures with a vascular network, addressing many challenges in regenerative medicine [120]. The presence of hepatic organoids fuels the prospect of implementing autologous organ transplants, where healthy patient liver tissues can be expanded and subsequently transplanted, thereby reducing the risk of adverse immune reactions [121].

Furthermore, exogenous cell therapy has emerged as an alternative to LT to understand and harness the regenerative capacity of hepatocytes and biliary epithelial cells, acting as stem cells to restore damaged epithelial populations. Identifying key signals can provide targeted therapeutic pathways and enable the development of therapies to enhance liver regeneration [74,122].

In 2013, Huch et al. pioneered the generation and implantation of bile duct-derived organoids in nude Balb/c mice, which differentiated into functional hepatocytes [123]. A study showed the use of extrahepatic cholangiocyte organoids for the reconstruction of the extrahepatic biliary tree in mice: ECOs transplanted into immunocompromised mice were observed to maintain gene expression and express key biliary markers, which allow for self-organization into bile duct-like tubes and repair of damaged biliary epithelia [99]. Subsequent studies demonstrated the high engraftment rate (80%) and survival (90 days) of liver organoids generated from both murine and human primary hepatocytes after transplantation into immunodeficient mice with damaged livers [124,125].

Cholangiocyte plasticity represents the strong point of this technique and potentially the future of regenerative medicine. Cholangiocytes have different transcriptional profiles, based on their location within the biliary tree, but the organoids lose these properties as they lack stimuli [120]. Following various local and environmental stimuli, it is possible to reconstitute the expression of specific markers and restore the different regional conformations [11]. Organoids derived from cholangiocytes have demonstrated the ability to regenerate up to 50% of the biliary tree in mice with injuries, and they have been successfully transplanted into ex situ perfused human livers, providing the first demonstration of the efficacy of organoids in regenerative medicine in human organs [8]. Sampaziotis et al. demonstrated that the transplantation of cholangiocytic organoids in a different region, compared to the original one, still allows the damage to be repaired. It was observed that the transplanted organoids (ICOs) at the level of the intrahepatic biliary tree formed a cell population made up of native and transplanted cholangiocytes, with the potential for regeneration of about 40–85%. As demonstrated, ICOs represent an important experimental tool for cholangiopathy pathogenesis investigation [126].

The development of cholangiocyte organoid systems can overcome the limitations of in vitro cholangiocyte cultures. There are significant challenges, as cells intended for transplantation must be highly functional to survive in hostile environments, such as bile, integrate into the vascular system, and engraft in the long term. Additionally, cell therapies are limited by the lack of integration with large-scale automated production platforms, but the use of robotic systems and bioreactors can overcome this limitation [127,128]. Finally, the use of a matrix, such as Matrigel, which can be potentially risky due to its chemically undefined nature [129], is driving the development of hydrogel matrices based on biological or synthetic polymers showing an acceptable safety profile, including genetic stability without the risk of carcinogenesis, to transition from clinical experimentation to practical applications [118].

The use of scRNA-seq (single-cell RNA sequencing) to characterize the transcriptional profile and phenotypic state of cells during regeneration can be crucial to solving another problem: understanding whether cholangiocytes undergo direct transdifferentiation or pass through an “intermediate progenitor” state after dedifferentiation [74]. Several recent studies have applied scRNA-seq to organoids to model organ development, tissue regeneration, and diseases. These studies demonstrate that the combination of these two cutting-edge technologies allows for the identification of rare or novel cell types and genetic markers in an organ, opening new perspectives in understanding and applying these technologies [122].

### 6.3. Challenges in Organoid Clinical Applications: From Bench to Bedside

There are several translational barriers, including regulatory, ethical, and technical challenges, which currently need to be addressed for the translation of organoid technology into clinical applications [130]. These challenges require a multidisciplinary approach among scientists, clinicians, ethicists, and regulatory agencies to develop standardized protocols, ameliorate culture techniques, and establish clear ethical guidelines. Table 3 resumes these multifaceted issues and recommends measures that need to be taken to ensure the clinical application of this technology. 

The potential of organoid-based approaches to improve patient care and outcomes in diverse disease settings poses regulatory and ethical issues [131]. Regulatory agencies are needed to define and implement guidelines and standards for the use of organoids in research and clinical applications through preclinical studies and clinical trials. Major ethical concerns are related to the use of human tissues or stem cells, development of an informed consent model for organoid donors, protection of donors’ identities and their personal information, and commercialization and patentability of organoids [132]. Moreover, the transplantation of organoids [131], use of gene editing [133], creation of chimeras [134], and long-term storage in biobanks [135,136] raise psychological and ethical concerns in society that should be considered and regulated to allow the bench-to-bedside translation of organoids.

Advances in culture techniques and biomaterials hold promise for overcoming these challenges and unlocking the full potential of organoid research. A major limitation is the maintenance of cellular functionality and viability over time due to culture conditions, such as media composition and growth factors; as the volume of organoids increases, so does the demand for nutrients and oxygen [130,137]. At the same time, the accumulation of toxic metabolites causes cell death and necrosis. The integration of a vascular system into organoids to allow the transport of nutrients and metabolic waste is important for recapitulating normal human physiological conditions and for long-term organoid culture [126,138,139]. This problem can be addressed using bioreactors that improve nutrient supply, and co-culturing organoids with endothelial cells or progenitors can promote vascularization during organoid formation. It has already been observed by Jin et al. [140] that vascularized hepatic organoids cultivated using chip technology have improved intercellular interactions and metabolic activity. It seems that the matrix, such as Matrigel, also influences organoid development, limiting their clinical applications, as it has a non-human origin, variable biochemical properties, and potential contamination risks [141,142,143]. Therefore, significant efforts are underway to develop synthetic matrices or hydrogels that are safer and more effective for organoid culture and their use in preclinical and therapeutic studies.

## 7. Conclusions

Biliary tract diseases, including primary sclerosing cholangitis, are a major cause of fibrosis, cirrhosis, and, in severe cases, LT. The lack of effective therapies for biliary diseases has led to the evaluation of new therapeutic options in the field of biliary regenerative medicine, which have the potential to radically change our management of these patients [144,145].

Significant advancements in the culture of hepatic and cholangiocyte organoids, along with in-depth regenerative medicine techniques, hold considerable promise for improving the lives of patients with advanced liver diseases. Cholangiocyte organoids represent a formidable technology for better understanding the molecular pathways underlying cholangiopathies and allows for the exploration of personalized therapeutic approaches for each patient and the repair of damaged biliary epithelia.

The application of organoids is still under investigation in humans, and there are still many challenges regarding the adaptation and translation of regenerative concepts and tools into truly regenerative therapies. This includes the development of reproducible methods for the composition and functionality of organoids and the enhancement of cellular maturation to ensure greater resemblance to pathophysiological processes. However, the results obtained to date are promising and represent a potential paradigm shift in the management of acute and chronic liver and biliary diseases [146].

## Figures and Tables

**Table 1:** The most common cholangiopathies.

	Incidence/ Prevalence	Epidemiology	Diagnosis
PBC	1–2 per 100,000 per year	60% Female	Autoimmune syndromes, AMA pos, ANA pos. No need of biopsy.
PSC	0–1.3 per 100,000 per year	60% Male	IBD, multiple, both intra- and extrahepatic strictures. No need of biopsy.
ISC	2.18 per 100,000	80% Male	Tissue IgG4 deposit, pancreatitis, sialadenitis. Sudden jaundice.

**Table 3:** Organoids from bench to bedside: regulatory, ethical, and technical challenges.

Issues/Challenges	Description
***Regulatory issues***	
Standardization	To establish standardized protocols for the generation, maintenance, and characterization of organoids, and ensure consistency and reproducibility.
Regulatory Approval	To demonstrate the safety, efficacy, and reliability of organoid-based therapies, regulatory agencies define guidelines and standards for the use of organoids in research and clinical applications.
Classification	Organoids may fall into regulatory gray areas, as they do not neatly fit into existing regulatory frameworks designed for traditional drugs or medical devices.
***Ethical issues***	
Source of Stem Cells	Organoids derived from induced pluripotent, embryonic, and adult stem cells raise ethical concerns about the source of these cells, consent, and privacy.
Informed Consent of Cell Donors	Ensure that donors of biological materials used to generate organoids provide informed consent. This includes understanding the purpose of the research, potential risks, and benefits involved.
Privacy and Data Security	To adhere to ethical guidelines regarding the use and sharing of sensitive data, ensuring the privacy and confidential information of donors and research data.
Creation of Chimeras	Organoid research may involve the creation of human–nonhuman chimeras, which raises ethical questions about the moral status and the boundaries between humans and other species.
Gene-Editing Tools	Unintended consequences and off-target effects can impact the safety and efficacy of gene-edited organoids. Ethical research practices should include a thorough evaluation of potential risks and long-term effects on both organoids and human health and ensure compliance with ethical standards and safety guidelines.
Organoid Transplantation	Off-the-shelf organoids of clinical-grade quality (size, degree of maturity, and functionality) to ensure safe clinical use.
Biobanking	To define the legal status of organoids for the governance of biobanks. Protection of the privacy and confidentiality of donors’ information and storage of sensitive biological data. To ensure the long-term sustainability of biobanks, implement best practices for sample preservation, and quality assurance and control.
***Technical Issues***	
Complexity and Heterogeneity	To Improve the structural and functional heterogeneity of organoids, to standardize their properties, and to better mimic the in vivo microenvironment of organs, recapitulating the complexity of their in vivo counterparts.
Scale-Up and Automation	Scaling-up organoid production and implementing automation for large-scale applications, such as high-throughput drug screening or transplantation therapies.
Functionality and Long-Term Stability	To optimize organoid culture conditions to increase functionality and ensure long-term stability and quality of organoids in in vitro setting. Vascularization is essential for organoid viability.

## Data Availability

For Literature Search Strategy, We performed a systematic literature search using the PubMed, SCOPUS, Web of Science, Google Scholar, and MedRxiv/BioRxiv (preprints) databases. We included scientific publications and preprint articles and book chapters. We screened all reference lists of the most pertinent studies in order to identify any missing publications. No new data were created or analyzed in this study. Data sharing is not applicable to this article.

## Abbreviations

Act A	Activin A
AdSCs	organ-restricted adult stem cells
AMAs	anti-mitochondrial antibodies
ANAs	specific anti-nuclear antibodies
AS	anastomotic stenosis
BA	biliary atresia
BCOs	organoids and cholangiocyte organoids derived from bile
BMP	bone morphogenic protein
BTPCs	biliary tree progenitor cells
CFTR	cystic fibrosis transmembrane conductance regulator
DAMPs	damage-associated molecular patterns
DCDs	donors after cardiac death
DR	ductular reaction
ECM	extracellular matrix
ECOs	extrahepatic cholangiocytes
EGF	epidermal growth factor
ELTR	liver transplant registry
ERCP	endoscopic retrograde cholangiopancreatography
ESCs	embryonic stem cells
FDOs	functional ductal organoids
FGF19	fibroblast growth factor 19
FSK	forskolin
GCOs	gallbladder cholangiocyte organoids
HGF	hepatocyte growth factor
HMP	hypothermic machine perfusion
HNF1β	hepatocyte nuclear factor 1β
HPC	hepatic progenitor cell
IBD	inflammatory bowel disease
IC	ischemic cholangiopathy
ICOs	intrahepatic cholangiocyte organoids
Ig	immunoglobulin
IL-6	interleukin-6
iPSCs	pluripotent stem cells
IRI	ischemia-reperfusion injury
ISC	IgC4-related sclerosing cholangitis
ITBLs	ischemic-type biliary lesions
LT	liver transplantation
MP	machine perfusion
NAS	non-anastomotic stenosis
NMP	normothermic machine perfusion
norUDCA	24-norursodeoxycholic acid
NOX	NADPH oxidase
NRP	normothermic regional perfusion
OCA	obeticholic acid
OOC	organ on a chip
OSM	oncostatin M
PBC	primary biliary cholangitis
PBGs	peribiliary glands
PDGFB	platelet-derived growth factor
PSC	primary sclerosing cholangitis
ROS	oxygen-containing reactive species
S1RPs	signal regulatory proteins
scRNA-seq	single-cell RNA sequencing
SOX9	SRY-box transcription factor 9
TGFβ	transforming growth factor beta
TNFα	tumor necrosis factor alpha
UDCA	ursodeoxycholic acid
